# Supercomputing modelling of advanced materials: preface

**DOI:** 10.1098/rsta.2022.0252

**Published:** 2023-07-10

**Authors:** C. Richard A. Catlow, Nora H. De Leeuw, Angelos Michaelides, Scott M. Woodley

**Affiliations:** ^1^ Department of Chemistry, University College London, 20 Gordon Street, London WC1H 0AJ, UK; ^2^ School of Chemistry, Cardiff University, Park Place, Cardiff CF10 1AT, UK; ^3^ School of Chemistry, University of Leeds, LS2 9JT Leeds, UK; ^4^ Department of Chemistry, University of Cambridge, Lensfield Road, Cambridge CB2 1EW, UK

The development and optimization of materials is critical to several contemporary scientific and technological challenges, including renewable energy technologies, novel catalytic routes to sustainable fuels and chemicals and electronic technologies. Computational modelling now plays an essential role in materials science and is used in an increasingly predictive manner. Simulations typically explore the multi-dimensional energy landscape, initially seeking low-energy minima, then the local ergodic region about these minima and the saddle points, or energy barriers, between them. The landscape is typically a function of atomic positions, and, as can be seen in figure 1, there may be a large number of atoms to include. Moreover, the problem size increases when the electronic structure is required, and a basis set or grid is used to describe the electron density. The use of a unit cell and periodic boundary conditions works well to address the system size problem for perfect crystalline systems. However, often properties are influenced by the effect of the defects, and a larger supercell or the removal of one or more of the periodic boundary conditions is needed to remove unwanted images of the defect. Alternatively, for modelling localized states, a non-periodic approach employing quantum mechanical/molecular mechanical (QM/MM) methods can prove very effective and such approaches are being further developed to enable simulations that have more than one QM region.

The power of modelling techniques continues to grow with the expansion of the capabilities of the computational hardware and the developments in algorithms and software. The growth of artificial intelligence (AI) and machine learning (ML) tools is also having a rapidly increasing impact on the field. In terms of hardware, nations are striving to upgrade their supercomputers from peta (10^15^) to exascale (10^18^ floating-point operations per second) and investigating the possibility of exploiting quantum computers. For example, the UK government has just confirmed around £900 million investment into exascale supercomputers and a dedicated AI research resource [1], and in preparation for the arrival of exascale supercomputers, the UK has invested £46M in the 5-year Excalibur Project [2] as larger supercomputers require significant investment in training [3] and software development, as current algorithms employed within materials software will not simply scale up due to the increase in communication between nodes [4].

In view of the rapid advances in materials modelling using high-performance computing, the Royal Society organized a Discussion Meeting in 2022 to review and discuss recent developments in the field, with a satellite meeting focusing on software solutions. The articles in this volume are based on a selection of the presentations at these meetings.

The topics discussed in the volume are wide ranging. Schön addresses the key area of structure prediction focusing on low-dimensional systems [5]. Two-dimensional materials are also explored by Lopez-Suarez *et al.* [6] who model how compression affects their mechanical and chemical properties. Nano-structured materials are discussed by van Speybroeck who focuses on their dynamical behaviour under operating conditions [7]. Christie discusses the challenges of modelling amorphous materials, including the application of large-scale dynamical simulations in modelling the effects of radiation damage [8]. The article also illustrates the growing role of ML-based potentials.

Modelling has for many years played an important role in understanding the processes of crystallization, which are the focus of two contributions [9–10]: Deymier *et al.* describe the modelling of the effect of stress on the recrystallization and dissolution of apatite—key processes in bone mineralization—while Rogal *et al*. describe how liquid structure and dynamics can provide information on crystal nucleation mechanisms. The breadth of the field is illustrated by the articles of Ahmad *et al.* [11] who describe the modelling of the adsorption of organic molecules on the surface of alumina which they show is relevant to the understanding of organic matter in soils, and by Gaikwad *et al.* [12] who employ steered molecular dynamics to model integrins—transmembrane adhesion proteins—with their work providing a model for integrin-mediated adhesion. Several contributions report significant technical advances. Savva *et al*. [13] discuss developments in kinetic Monte Carlo techniques used in modelling chemical kinetics and of growing importance in computational catalysis. Woods *et al*. [14] present developments in the field of discrete Markov chains of importance not just in physical but also in life and social sciences. Blake *et al.* [15] describe the development of a consistent set of forcefields for modelling aqueous metal carbonates—a topic again of relevance to understanding crystallization—and they show how large-scale simulations are facilitated using GPU technology. Finally, Guan *et al.* [16] report new developments allowing the accurate calculation of both infrared and Raman spectra using a QM/MM approach—a capability of value in both catalytic and biomolecular sciences.

**Figure 1.**
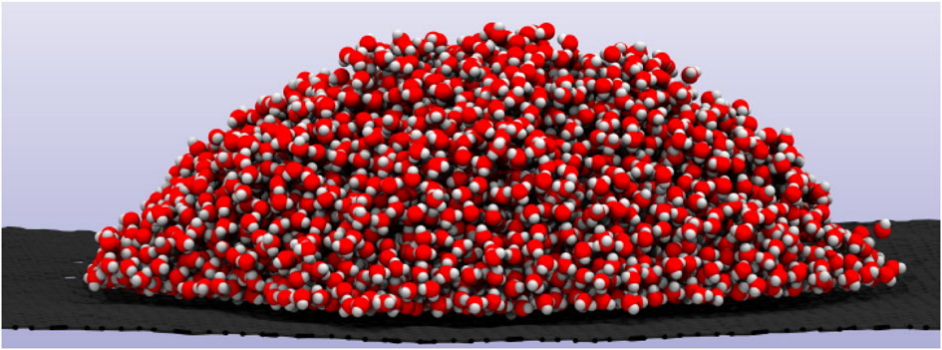
A minuscule water droplet on a surface using a stick and ball model for the water molecules; for a 3 g droplet, there will be approximately 10^23^ water molecules. Image obtained from machine learning accelerated simulations by Dr Christoph Schran, University of Cambridge.

We hope that the articles in the volume illustrate both the achievements and challenges in the field of materials modelling using high-performance computing, and that they convey the excitement of the field and the pace at which it is evolving.

Finally, we would like to thank both the Royal Society Scientific Meetings and Publishing staff for their help and support in the organization of both the Discussion and Satellite meeting and the preparation of this volume.

## Data Availability

This article has no additional data.
